# Preschool Anxiety Disorders Predict Different Patterns of Amygdala-Prefrontal Connectivity at School-Age

**DOI:** 10.1371/journal.pone.0116854

**Published:** 2015-01-27

**Authors:** Kimberly L. H. Carpenter, Adrian Angold, Nan-Kuei Chen, William E. Copeland, Pooja Gaur, Kevin Pelphrey, Allen W. Song, Helen L. Egger

**Affiliations:** 1 Duke University Medical Center, Center for Developmental Epidemiology, Department of Psychiatry and Behavioral Sciences, Durham, North Carolina, United States of America; 2 Duke University Medical Center, Brain Imaging and Analysis Center, Durham, North Carolina, United States of America; 3 Vanderbilt University School of Medicine, Chemical and Physical Biology, Nashville, Tennessee, United States of America; 4 Yale University, Yale Child Study Center, New Haven, Connecticut, United States of America; Technion - Israel Institute of Technology, ISRAEL

## Abstract

**Objective:**

In this prospective, longitudinal study of young children, we examined whether a history of preschool generalized anxiety, separation anxiety, and/or social phobia is associated with amygdala-prefrontal dysregulation at school-age. As an exploratory analysis, we investigated whether distinct anxiety disorders differ in the patterns of this amygdala-prefrontal dysregulation.

**Methods:**

Participants were children taking part in a 5-year study of early childhood brain development and anxiety disorders. Preschool symptoms of generalized anxiety, separation anxiety, and social phobia were assessed with the Preschool Age Psychiatric Assessment (PAPA) in the first wave of the study when the children were between 2 and 5 years old. The PAPA was repeated at age 6. We conducted functional MRIs when the children were 5.5 to 9.5 year old to assess neural responses to viewing of angry and fearful faces.

**Results:**

A history of preschool social phobia predicted less school-age functional connectivity between the amygdala and the ventral prefrontal cortices to angry faces. Preschool generalized anxiety predicted less functional connectivity between the amygdala and dorsal prefrontal cortices in response to fearful faces. Finally, a history of preschool separation anxiety predicted less school-age functional connectivity between the amygdala and the ventral prefrontal cortices to angry faces and greater school-age functional connectivity between the amygdala and dorsal prefrontal cortices to angry faces.

**Conclusions:**

Our results suggest that there are enduring neurobiological effects associated with a history of preschool anxiety, which occur over-and-above the effect of subsequent emotional symptoms. Our results also provide preliminary evidence for the neurobiological differentiation of specific preschool anxiety disorders.

## Introduction

A number of community studies have shown that clinically significant anxiety can be identified and diagnosed in children as young as 2 years of age [1–13]. These anxiety symptoms are associated with significant impairment [3, 4] and predict later psychopathology and impairment [2–4, 14–18]. Furthermore, recent research has shown that anxiety in early childhood differentiates into phenotypically distinct subtypes similar to patterns of anxiety seen in later adolescence and adulthood [19–23]. These subtypes include social phobia, separation anxiety, and generalized anxiety. These disorders are among the earliest occurring and most common psychiatric disorders in young children [3, 4] and occur at rates in early childhood that are similar to rates in later childhood [24]. The anxious distress characteristic of these anxiety disorders is associated with difficulty with emotion regulation processes that develop over toddlerhood, with self-regulatory processes apparent around 7–8 months and engagement of emotion regulation strategies emerging by 2 years and continuing to develop over the preschool period and beyond [25, 26]. The emergence of these emotion regulation capabilities is associated with the development of prefrontal-limbic connections, with better emotion regulation capabilities in young children correlated with both increased dorsomedial and decreased ventromedial prefrontal cortex activity [27, 28]. Furthermore, difficulty with emotion regulation processes, which are characteristic of anxiety disorders, has been linked to disruption of interactions between the amygdala and portions of the prefrontal cortex [29–31].

Phenotypically, generalized anxiety, separation anxiety, and social phobia share the common feature of anxious distress; however, associated characteristics differ between disorders. Social phobia and separation anxiety are characterized by fear and avoidance of specific types of social situations and stimuli [32, 33].

Generalized anxiety is characterized by pervasive and intrusive worry about a range of situations and stimuli that may be in the past, present, and/or future [24, 31]. Previous studies have suggested that the phenotypic differences between anxiety disorders may reflect different patterns of amygdala-prefrontal cortex dysregulation [30, 31, 34]. Studies have shown that different amygdala-prefrontal networks are associated with ruminative worry and with fear and avoidance. Amygdala-*dorsal* prefrontal neural networks are associated with the cognitive regulation of emotion and are linked to both normative and pathological worry [35, 36]. Amygdala-*ventral* prefrontal networks are involved in more automatic emotion regulation and are associated with the fear response [37–40]. In support of this worry-fear distinction, amygdala hyperactivation and dysregulation of amygdala-ventral prefrontal fear networks has been reported in both social phobia and adult anxious attachment, which shares phenotypic similarities with separation anxiety disorder [32, 41–43]. Amygdala findings in generalized anxiety are more heterogeneous [44–50], although there have been consistent reports of dysregulation of both amygdala-prefrontal connectivity and of regions associated with worry, including the dorsomedial prefrontal and anterior cingulate cortices [35, 36].

While fear and avoidance of social situations and stimuli are core characteristics of both disorders, social phobia and separation anxiety are also phenotypically distinct. Pine and colleagues have noted similarities between the hypervigilance to social information that characterizes social phobia and the conditioned fear response, which is associated with interactions between the amygdala and ventral prefrontal cortex [30, 32]. Neuroimaging studies have found dysregulation of the ventral prefrontal cortex and aberrant amygdala-ventral prefrontal cortex connectivity in social phobia [32, 43]. Although there is a paucity of neuroimaging studies of separation anxiety, anxious attachment styles in adulthood are associated with both greater amygdala and less orbitofrontal cortex activation [41, 42]. Physiological studies have reported greater hypothalamic-pituitary-adrenal (HPA) axis activity in children with separation anxiety [51]. The orbitofrontal cortex, amygdala, and associated lateral prefrontal cortex regulate the HPA axis and non-human primate studies implicate this network in the neural response to separation distress [40, 52]. Thus, although amygdala hyperactivation is associated with both social phobia and separation anxiety, the phenotypic differences between the disorders may be a result of dysregulation of different ventral prefrontal networks: the ventromedial prefrontal fear-conditioning network in social phobia and the orbitofrontal and lateral prefrontal HPA axis regulatory network in separation anxiety.

Most of the neuroimaging studies on anxiety disorders are cross-sectional with evaluation of the neural correlates of concurrent anxiety disorders. A limitation of cross-sectional studies is that we cannot disentangle the degree to which neurobiological findings represent state-dependent brain differences (i.e. when an individual has a disorder, they also have brain changes, but the brain returns to normal when the disorder resolves) or represent a trait or developmental process that presented early in life and has persisted whether or not the earlier symptomatology persists. Understanding remitting versus enduring neural effects of impairing anxiety in early childhood may enable us to identify early childhood biomarkers of risk for impairing anxiety and depression across the lifespan and to target our interventions or preventive efforts as early as possible. To our knowledge, our study is the first longitudinal study to examine the relationship between impairing anxiety in the preschool period and differences in neural correlates at school age. Additionally, few studies have explored the neural correlates of distinct anxiety disorders while controlling for between-anxiety comorbidity which is relatively common. In our study 30% of the anxious preschoolers had two or more anxiety disorders [53], a rate which is consistent with previous reports [14, 54]. To address both (1) the enduring neural effects associated with preschool anxiety disorders and (2) the neurobiological differences between these disorders, we need prospective, longitudinal neuroimaging studies that include children with a variety of anxiety disorders and are designed to examine anxiety symptoms and the comorbidity between anxiety and non-anxiety disorders across this developmental period.

In this paper we present the first wave of functional magnetic resonance imaging (fMRI) data from a prospective, longitudinal imaging study of the neural underpinnings of early childhood anxiety. The children in the imaging study were recruited from a large, community-based study of anxiety in preschool children. We hypothesized that impairing anxiety in the preschool period would be associated with differences in amygdala-prefrontal cortex networks at school age and that these differences would persist when controlling for school-age symptoms. While we can identify impairing anxiety in children two to five years old, we have found that five and a half is the youngest age for consistent success in conducting fMRI scans. By using preschool anxiety symptoms to predict neural differences at school-age that occurs over-and-above school-age symptomatology, we aimed to identify early brain-level differences or a “neural signature” linked to clinically significant anxiety in young children. A secondary, exploratory hypothesis of this study was that amygdala-prefrontal network differences would vary with the distinct phenotypic characteristics of these preschool anxiety disorders and that these differences would mirror the disorder specific dorsal-ventral patterns described in previous studies.

## Methods

### Study Design

Children were recruited from the Duke Preschool Anxiety Study, a cross sectional, screen-stratified study of anxiety in children ages two to five years old. The Duke Preschool Anxiety Study was a three part study, including a screening phase (N = 3,433), an in home assessment phase (N = 917), and a case-control laboratory phase (N = 502) [53]. Parents completed the Preschool Age Psychiatric Assessment (PAPA) in the in-home phase of the Preschool Anxiety Study when their children were ages 2 to 5 years old [12]. Children who met criteria for impairing generalized anxiety disorder, social phobia, and/or separation anxiety disorder were recruited as “cases” for the laboratory phase (n = 254). A random sample of 248 children who did not meet criteria for an anxiety disorder was recruited as our comparison group. Children in the laboratory phase were not excluded for comorbid non-anxiety disorders or for taking psychotropic medications (one child was on Zoloft).

**Figure 1:**
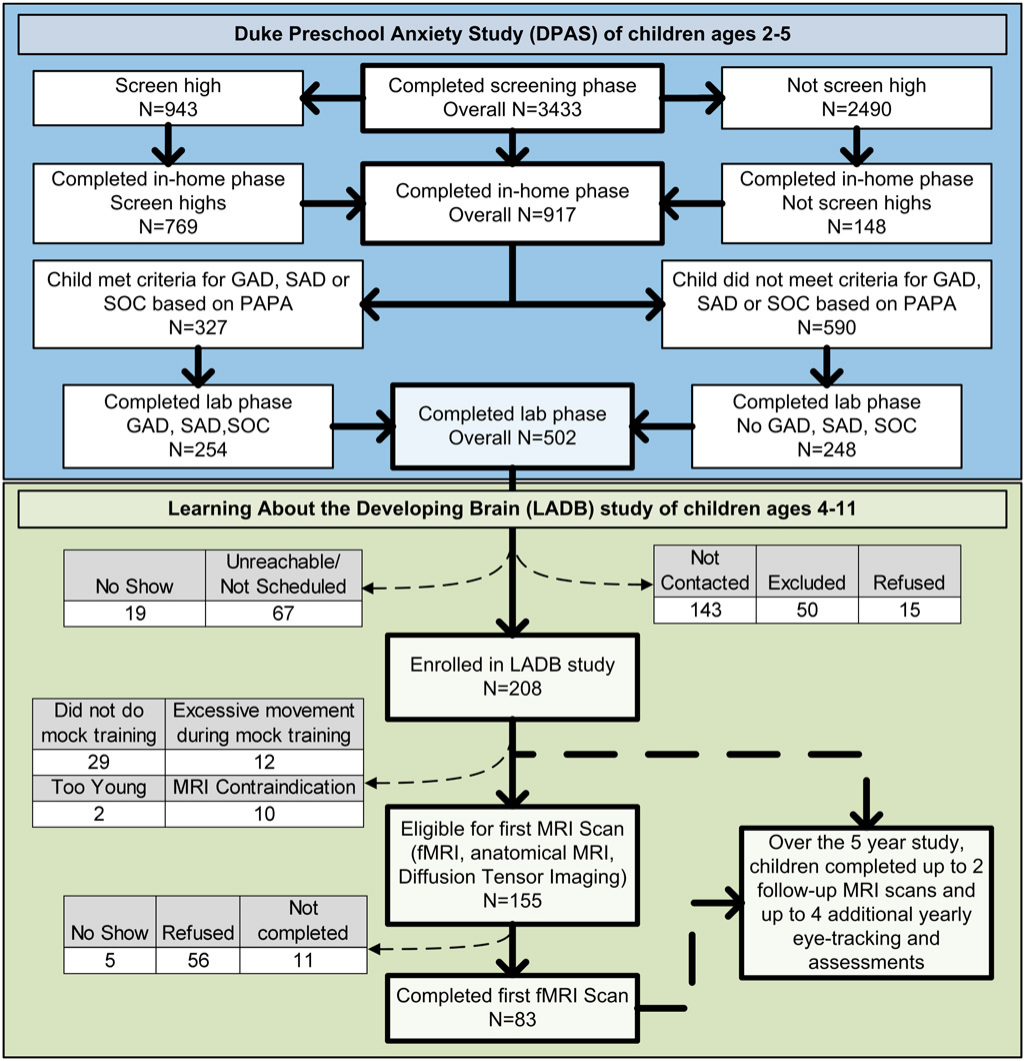
Sampling design of the Preschool Anxiety and Learning About the Developing Brain studies. See S1 Text for details. Key: GAD = Generalized Anxiety Disorder; SAD = Separation Anxiety Disorder; SOC = Social Phobia; PAPA = Preschool Age Psychiatry Assessment.

In the Learning about the Developing Brain study (LADB), 208 of the 502 children who participated in the case-control laboratory phase of the Preschool Anxiety Study were recruited to take part in a five year prospective, longitudinal eye tracking and neuroimaging study of early childhood brain development and anxiety disorders. Fig. 1 depicts the design of these two linked studies. In the first wave of the LADB study, 155 children were eligible to participate in the imaging phase. Inclusion criteria are detailed below. This paper reports on the fMRI data collected in this first wave of the LADB study. Descriptions of the subsequent waves of LADB data collection are summarized in S1 Text.

### Measures of Early Childhood Psychopathology

The PAPA was administered in the Preschool Anxiety Study when the children were two to five years old and in the LADB study when the children were six years old. The PAPA is a parent-report instrument for the assessment of psychopathology in 2–5-year-olds and is based on the parent version of the Child and Adolescent Psychiatric Assessment [55]. The PAPA uses a highly structured protocol, with requires questions and probes, however, the onus throughout is on the interviewer to ensure that interviewees (1) understand the question being asked; (2) provide clear information on behavior or feelings relevant to the symptom; and (3) report the symptom at a pre-specified level of severity as defined in an extensive glossary. When symptoms are reported, their frequency, duration and dates of onset are collected, to determine whether they meet the symptom and duration criteria for the various DSM diagnoses. A three month “primary period” is used, because shorter recall periods are associated with more accurate recall [56]. The PAPA includes assessment of most DSM-IV diagnostic criteria insofar as they are relevant to younger children, plus all items in the Diagnostic Classification: 0–3R [57]. DSM-IV diagnoses include: attention-deficit/hyperactivity disorder, oppositional defiant disorder, conduct disorder, depression (major depression, dysthymia and depression-not otherwise specified), anxiety disorders (separation anxiety disorder, generalized anxiety disorder, social phobia, specific phobia, posttraumatic stress disorder, and selective mutism), and elimination disorder (enuresis and encopresis). The assessment of impairment resulting from each group of symptoms was based upon the World Health Organization’s International Classification of Functioning, Disability and Health [58]. To avoid tapping into normative fears, impairment from anxiety was required for all anxiety diagnoses. A study of the test-retest reliabilities of the PAPA concluded that the diagnostic reliability of the PAPA is on a par with those achieved by older child, adolescent and adult psychiatric interviews [12].

The variables used in this paper include impairing generalized anxiety, social phobia, and separation anxiety disorders assessed when the children were preschoolers (ages 2–5 years old). A composite score of generalized anxiety, social phobia, separation anxiety, and depression symptom counts, obtained from the PAPA that was completed when the children were 6 years old, was used as a measure of school-age emotional symptomatology.

### Participants

Children were eligible for an MRI in the first wave of the LADB study if they (1) completed a laboratory assessment in the Preschool Anxiety Study, (2) were at least five and a half years old during the recruitment period, and (3) successfully completed a mock scanner training session, detailed in S1 Text. In this paper, “anxious children” are those who met criteria for an impairing anxiety disorder as preschoolers; “non-anxious children” are those who did not meet criteria for an anxiety disorder as preschoolers. In total, 155 children were eligible for the MRI scan in this first wave. Of these children, 56 parents or children refused to participate; five did not show up for multiple MRI appointments; and 11 children asked to be removed from the scanner before data could be collected. A total of 83 children completed a scan, of which 41 had met criteria for an anxiety disorder as a preschooler. Anxious children were less likely to complete an MRI scan (c^2^ = 7.25, p>0.01), however the group of anxious children who completed a scan were similar to children who did not complete the scan in their number of preschool anxiety symptoms (t(91) = 0.13, p = 0.9) and impairments (t(91) = -0.29, p = 0.8). Of the remaining 83 children who completed the fMRI scan, 45 (54%) children had usable data. Of participants without usable data, 29 had excessive motion, defined by either a signal-to-noise ratio ≤60 or movement >1 voxel in any direction as measured with in-house quality control software and motion parameters produced by FSL FLIRT [59, 60]; 5 fell asleep; and 4 had other reasons for not producing usable data (e.g. scanner error). Children with lower IQs (t(81) = -2.10, p = 0.04) were less likely to have usable fMRI data. There were no other significant differences between the groups of children with and without usable data. Both groups were similar in age (t(81) = -0.63, p = 0.53), sex (c^2^=1.19, p = 0.28), race (c^2^=3.22, p = 0.07), and preschool anxiety status (c^2^ = 0.12, p = 0.73). There were no differences in any motion parameters (Rotation: t(43) = 0.69, p = 0.49; Translation: t(43) = 1.69, p = 0.1; Displacement: t(43) = 1.07, p = 0.29) between anxious and non-anxious children.

Table 1 summarizes the demographic and clinical characteristics of the 22 anxious and 23 non-anxious children, ages 5.5 to 9.5 years old, who had usable fMRI data in this first wave of data collection. As preschoolers, the children in the anxious group met criteria for impairing generalized anxiety, separation anxiety, social phobia, or a combination of these disorders. In total, 15 children as preschoolers met criteria for generalized anxiety, 10 for separation anxiety, and 11 for social phobia. Twelve of the 22 anxious children met criteria for more than one anxiety disorder. Despite between-anxiety comorbidity, there was no significant correlation between generalized anxiety and separation anxiety (Φ = -0.16, p = 0.5) or between separation anxiety and social phobia (Φ = 0.00, p = 1.0). There was a significant correlation between generalized anxiety and social phobia, however it was a significant negative correlation (Φ = -0.49, p = 0.02), suggesting that children were less likely to meet criteria for generalized anxiety if they met criteria for social phobia and vice versa. Anxious children were, on average, a year younger than non-anxious children (t(43) = -2.37, p = 0.02; Table 1). To ensure our results were not associated with this difference in age we ran all models both with and without age as a covariate and the findings remained the same. Anxious children were similar to non-anxious children in race (c^2^ = 0.203, p = 0.7), sex (c^2^ = 1.29, p = 0.3), handedness (p = 0.6, 2-tailed FET), poverty status, which was used as a proxy for SES (p = 0.72, 2-tailed FET), and IQ (t(43) = -0.16, p = 0.9).

**Table 1:** Sample Characteristics.

		**Non-Anxious**	**All Anxious**	**Generalized Anxiety^†^**	**Separation Anxiety^†^**	**Social Phobia^†^**
		**(N = 23)**	**(N = 22)**	**(N = 15)**	**(N = 10)**	**(N = 11)**
Demographic Variables	African American	12	10	8	6	3
	Female	13	16	12	7	8
	Right Handed	16	18	14	7	8
	Federal income below federal poverty line	4	6	5	5	2
	Age at Scan^a^	7.4(0.94)	6.72(1)*	6.72(1.02)*	6.87(1.28)	6.5(0.86)*
	IQ^a^	104.5(14.03)	103.86(10.82)	103.53(11.51)	103.2(10.63)	106.18(9.54)
	Impairment^††^ (range: 0–10)^a^	0.74(1.09)	3.5(2.35)*	3.93(2.66)*	3.8(2.62)*	3.27(1.68)*
	Preschool emotional symptoms (range: 0–9)^a^	2.17(1.99)	6.54(2.91)*	7.27(3.13)*	8.4(2.91)*	5.82(2.4)*
	School-Age emotional symptoms (range 0–14)^a^	2.43(1.95)	4.14(3.38)*	4.67(3.89)*	4.8(4.39)*	2.91(1.87)
Preschool Comorbidity	No disorder	20	-	-	-	-
	Generalized Anxiety Only	-	6	6	-	-
	Separation Anxiety Only	-	1	-	1	-
	Social Phobia Only	-	3	-	-	3
	Generalized Anxiety + Separation Anxiety	-	4	4	4	-
	Generalized Anxiety + Social Phobia	-	3	3	-	3
	Separation Anxiety + Social Phobia	-	3	-	3	3
	All three anxiety disorders	-	2	2	2	2
	Depression	1	3	3	1	1
	Other Diagnoses	2	10	9	5	4
School-Age Diagnosis	Anxiety Disorder(s)	4	11	6	5	6
	No Anxiety Disorder	19	11	9	5	5
	Other Diagnoses	1	3	2	3	1

Notes: Other diagnoses include conduct disorder, oppositional defiant disorder, and ADHD.

*Significant difference from non-anxious children at p<0.05;

^a^The values in these columns represent average (standard deviation);

^†^These are not mutually exclusive groups;

^††^This represents the level to which psychiatric symptoms interfere with everyday functioning.

Table 1 also details preschool comorbidity between the anxiety disorders and depression and other disorders, as well as the school-age disorders for each group. Half of the children who met criteria for an anxiety disorder as preschoolers still met criteria for an anxiety disorder at school-age, and 4 children who did not meet criteria for an anxiety disorder as preschoolers, did meet criteria for an anxiety disorder at school age. As noted above, we measured school-age emotional symptoms using a composite anxiety and depression symptom scale.

The Duke University Medical Center Institutional Review Board approved this study. Verbal assent from the child and written informed consent from the parent was obtained following a complete description of the study. The primary caregiver/parent was reimbursed $40 for each laboratory visit and $75 for each MRI visit. Additionally, the child chose a toy worth approximately $5 for participation in the laboratory portion of the study and a toy worth approximately $10 for participation in the MRI study. Children also received a frame at the MRI visit and were sent pictures of their brain that they could place in the frame, along with a thank you card, after their visit. Participants were given vouchers to cover parking cost.

### Functional MRI Task

Each subject completed two runs of a block-design, emotional face-processing task. Thirty-six face stimuli expressing angry, fearful, happy, or neutral emotions were selected from the NimStim Stimulus Set [61]. The current manuscript focuses on results from the angry and fearful faces, as these have been most consistently reported to elicit differences in previous studies in adults and adolescents with anxiety disorders. Future work will explore differences in response to both happy and neutral faces. Furthermore, many previous studies have used neutral faces as a baseline condition. However, we did not choose this approach due to evidence suggesting that neutral faces are not processed the same in social phobia as they are in unaffected individuals [62]. Each run began and ended with a 16-second fixation block. 15-second task blocks were separated by 12-second baseline fixation blocks, consisting of a centralized solid colored star. Each face-block contained 12 pictures from a single emotion category. Faces were presented for 1.25 seconds with no interstimulus interval. Each run consisted of three blocks of each emotion type, with the order of the emotion categories randomized. The paradigm was programmed in CIGAL [63], projected onto a screen at the back of the scanner bore, and viewed via an angled mirror mounted on the scanner headcoil. To keep the child engaged and enable us to measure task compliance, the child was instructed to press a button in response to a single face wearing glasses within each block. This face was randomly located within the block and expressed the same emotion as other pictures within the block. Average task accuracy was reasonable (non-anxious: 83.33%; anxious: 82.29%) and similar between the groups (t(36) = -0.15, p = 0.88).

### MRI Acquisition

Fifteen participants (8 anxious, 7 non-anxious) were scanned on a 3T GE Signa EXCITE HD system and thirty participants (14 anxious, 16 non-anxious) were scanned on a 3T GE MR750 system. Children run on different scanners were similar in age (t(43) = -0.95, p = 0.3), race (c^2^ = 1.11, p = 0.3), sex (c^2^ = 2.38, p = 0.1), IQ (t(43) = 0.13, p = 0.9), and anxiety status (c^2^ = .0.18, p = 0.6). The same pulse sequences and parameters were used on both scanners and identical scanner performance, including spatial accuracy and dynamic signal stability, was confirmed through calibration experiments in an agar phantom. Each functional scan lasted 5 minutes and 44 seconds, over which 172 functional images were acquired. The task was triggered by a scanner pulse following an 8 second delay included to allow for scanner stabilization. Within each run, 34–39 slices were acquired parallel to the AC-PC plane using a BOLD-sensitive EPI sequence (Voxel size: 4mm^3^; Repetition time: 2000ms; Echo time: 27ms; Field-of-view: 24cm; Flip-angle: 77; Interleaved-odd acquisition). A high-resolution T1-weighted anatomical scan was acquired for co-registration with the functional images using a 3D-FSPGR sequence with SENSE (Voxel size: 1mm^3^; Repetition time: 8.096ms; Echo time: 3.18ms; Inversion time: 450ms; Field-of-view: 25.6cm; Interleaved-odd acquisition). Scanner was included as a covariate in all analyses despite evidence suggesting that differences between scanners of similar manufacturer and field strength are relatively minor.

### Analytic Approach

**Preprocessing and Whole Brain Analysis.** Data were analyzed with FSL version 5.98 using standard procedures. Volumes with motion and intensity jumps greater than three standard deviations from the run mean were flagged for removal as part of a modified scrubbing protocol [64]. In the case of intensity jumps, run means were determined as the absolute deviation relative to the run mean after each voxel’s data was passed through a (1/60)Hz high-pass filter to eliminate low-frequency drift. Entire task blocks were excluded if (i) two volumes were removed from the beginning of the block or (ii) more than 3 volumes in total were removed from the block. Additionally, the entire run was excluded from subsequent analyses if more than one block of an emotion condition was removed. On average, 8 volumes were removed from the non-anxious group’s data and 4 volumes were removed from the anxious group’s data. There were no differences in the amount of data that was removed through scrubbing between the anxious and the non-anxious groups (t(64) = -1.7, p = 0.09). After scrubbing, non-brain tissue was removed using FSL’s Brain Extraction Tool [65]. Motion correction was analyzed by center-of-mass measurements in three orthogonal planes using FSL MCFLIRT [59, 60]. The images were then corrected for slice timing and spatially smoothed using a Gaussian kernel of full-width half-maximum of 5mm. Data were acquired in an interleaved-odd pattern, thus we utilized the FSL default interleaved option for slice timing correction. Finally, images were high-pass filtered, then were normalized into a common pediatric atlas space [66] representing children ages 4.5–8.5 (42/45 of our subjects fell within this age range) in a step-wise fashion using FSL’s FLIRT.

FSL’s FEAT was used for the whole-brain analysis. The onset timing for each face block was convolved with a double-gamma hemodynamic response to create a regressor of the predicted neural response to face blocks. A mixed effects general linear model (GLM) was used to identify regions where the entire sample, regardless of group membership, activated in response to faces. Multiple comparisons in the whole-brain analysis were controlled for with a GRF-theory cluster-corrected threshold of p < 0.05 and a conservative z-statistic threshold of 3.3 (p< 0.001).

**Region of Interest Analyses.** Because of a priori hypotheses, we focused on defined regions of interest (ROIs) and the angry and fearful face stimuli. S1 Fig. depicts the location of these ROIs. ROIs consisted of 6mm spheres formed around coordinates from meta-analyses of the neural bases of face-processing, emotion perception, emotion regulation, and threat appraisal [67–70]. We chose to use a coordinate based system for drawing our ROIs, as opposed to anatomical segmentations, in an effort to constrain our analyses to focus on only those portions of what are often large anatomical regions, particularly in the prefrontal cortex, that have been implicated in our constructs of interest. Spheres were created on the template brain around these coordinates and then transformed into subject space using FSL FLIRT [59, 60]. Maximum percent signal change within an ROI was extracted with the FSL Featquery tool at the subject level for each condition within a run. Differences in activation within specific ROIs were tested with SAS mixed multiple regression models, with run and condition modeled as repeated measures and standard errors and test statistics of fixed-effect parameters adjusted using the empirical option. Two separate types of mixed models were run for each ROI: (A) “simple model,” comparing children without a history of anxiety as preschoolers to an ‘all anxious’ group or to one anxiety disorder at a time, and (B) “comorbidity model,” which simultaneously modeled dichotomous variables representing preschool diagnoses of generalized anxiety, separation anxiety and social phobia. Scanner, age at scan, sex, race, IQ, and school-age emotional symptoms, were included as covariates in all ROI analyses. School-age emotional symptoms were measured with the follow-up PAPA and include symptom counts for generalized anxiety, separation anxiety, social phobia, and depression, which is often comorbid with, and shares commonalities with, anxiety disorders.

We employed statistical controls to account for comorbidity in our study. Specifically, we used an additive main effect model, which provides marginal predictions for each anxiety disorder on brain activation. This approach accounts for non-independence between groups and identifies the unique contribution of each anxiety disorder in the presence of other anxiety disorders (i.e. controls for comorbid disorders). As such, by including all diagnoses within the same linear model, while simultaneously controlling for the effects of current symptomatology, we were able to identify the **specific contribution** of each individual preschool diagnosis, over-and-above the effect of current symptomatology.

**Psychophysiological Interaction Analysis.** A psychophysiological interaction (PPI) analysis was used to investigate group differences in task-based functional connectivity between the right and left amygdala and the rest of the brain. The PPI analysis was implemented in FEAT using standard procedures [71]. Specifically, the timecourse was extracted from right and left amygdala ROIs for each subject. The PPI utilized mixed effects GLM to explore the interaction between the amygdala time course (physiological regressor), and a psychological regressor comprised of the timing of the onset of either angry or fearful face blocks (analyzed separately), convolved with a double-gamma hemodynamic response function where the baseline was the blocks of colored stars. Regressors consisting of the timing for each of the other task conditions, scanner, and school-age symptom counts were included as covariates. Similar to the ROI analyses, comorbidity was modeled by simultaneously including regressors for generalized anxiety, separation anxiety, and social phobia. By including regressors for all three disorders in the same model, the GLM accounts for variance shared between the disorders, allowing us to detect only those difference that are specific to each disorder and occur over-and-above the shared effects. Multiple comparisons were accounted for in the PPI analyses with a GRF-theory cluster-corrected threshold of p<0.05 and a z-statistic threshold of 1.96 (p≤0.05). After thresholding, group comparison analyses were restricted to clusters identified in the main effect map from the comparison group (e.g. the non-anxious>generalized anxiety comparison was masked by the main effect of the non-anxious group and the generalized anxiety>non-anxious comparison was masked by the main effect of the generalized anxiety group). This ensured that between group differences represent differences in regions that are significantly connected to the amygdala in the comparison group.

### Anatomical Region Identification

For both the whole brain and the PPI analyses, regions displaying significant effects were identified using the Harvard-Oxford cortical and subcortical probabilistic atlases [72–75], included in the FSL Analysis package. Additionally, when available, Brodmann Area (BA) designations were determined using the Talairach atlas [76–78], also included in the FSL Analysis package.

## Results

### Whole Brain and Region of Interest Analyses

To confirm there was no residual motion or data scrubbing artifacts, we first ran a whole brain analysis collapsing across all subjects and face blocks. S2 Fig. demonstrates robust activation across the face-processing network, including the amygdala, fusiform gyrus, and prefrontal cortex.

Table 2 and S3 Fig. summarize the results from the ROI analyses. All ROI analyses represent differences in response to either angry or fearful faces as compared to the baseline star condition. Compared to children without a preschool anxiety disorder, children with a preschool anxiety had significantly less left dorsolateral prefrontal cortex activity in response to angry faces (F(1,37) = 4.47, p = 0.0413). There were no other significant differences in the prefrontal cortex to either face condition, nor were there differences in amygdala activation to either face condition. Although not a focus of the current study, S1 Table summarizes the results from the comparisons between the children without a preschool anxiety disorder and each individual preschool anxiety disorder.

**Table 2:** Percent signal change and mixed model results from ROI analyses.

**Regions**			**MNI Coordinates**	**Average Max Percent Signal Change (Standard Deviation)**					**Comorbidity Model Adjusted p-Values**				**Any Anx vs Non-Anx (p-values)**
				**Non-Anx**	**Any Anx**	GAD*	SAD*	SoPh*	**GAD**	**SAD**	**SoPh**	**Current**	
**Angry Faces**	Amygdala	L	-20, -6, -15	0.88(0.68)	0.8(0.52)	0.85(0.52)	0.8(0.65)	0.69(0.42)	0.697	0.847	0.068	0.264	0.398
		R	20, -4, -15	1.12(0.77)	0.9(0.72)	0.93(0.7)	0.95(0.82)	0.98(0.77)	0.334	0.613	0.4	0.159	0.147
	vmPFC	M	0, 38, -18	2.46(1.86)	2.01(1.87)	2.03(1.78)	2.22(2.34)	1.85(1.92)	0.932	0.573	0.745	0.521	0.688
		R	6, 40, -22	3.18(2.71)	3.06(2.87)	3.17(2.92)	2.93(2.85)	2.54(2.99)	0.98	0.809	0.492	0.216	0.522
	Lateral OFC	L	-24, 28, -14	1.18(1.1)	1.04(0.86)	1.11(0.82)	0.72(0.66)	0.75(0.81)	0.536	0.171	0.359	0.291	0.787
		R	26, 24, -22	2.7(2.03)	2.76(2.24)	2.98(2.32)	3.05(2.49)	2.04(1.88)	0.67	0.412	0.069	0.582	0.9
	dlPFC	L	-42, 13, 27	0.47(0.39)	0.34(0.27)	0.36(0.29)	0.3(0.28)	0.28(0.25)	0.197	0.63	0.003	0.255	0.041
		R	48, 17, 29	0.99(0.93)	0.95(1.3)	1.11(1.5)	0.52(0.48)	0.65(0.45)	0.516	0.015	0.343	0.461	0.755
	vlPFC	L	-42, 25, 3	0.62(0.53)	0.51(0.51)	0.54(0.58)	0.67(0.69)	0.43(0.35)	0.741	0.095	0.087	0.598	0.827
		R	42, 25, 3	0.56(0.43)	0.51(0.36)	0.5(0.41)	0.44(0.46)	0.48(0.25)	0.674	0.636	0.313	0.565	0.499
	rACC	R	4, 47, 7	0.48(0.56)	0.46(0.55)	0.39(0.53)	0.36(0.58)	0.56(0.55)	0.481	0.489	0.598	0.579	0.727
	dmPFC	R	10, 54, 18	0.48(0.36)	0.57(0.57)	0.53(0.56)	0.47(0.65)	0.82(0.53)	0.255	0.035	0.001	0.103	0.586
**Fearful Faces**	Amygdala	L	-20, -6, -15	0.7(0.6)	0.87(0.77)	1.03(0.85)	0.89(0.96)	0.68(0.53)	0.035	0.509	0.563	0.978	0.135
		R	20, -4, -15	0.96(0.7)	1(0.74)	1.13(0.76)	1.16(0.96)	1.11(0.78)	0.262	0.407	0.569	0.137	0.613
	vmPFC	M	0, 38, -18	2.28(1.9)	1.54(1.4)	1.21(1.01)	1.34(1.13)	2.14(1.6)	0.017	0.032	0.04	0.071	0.228
		R	6, 40, -22	3.42(2.9)	2.29(2)	2.14(2.09)	2.17(2.39)	2.72(1.84)	0.391	0.346	0.29	0.211	0.309
	Lateral OFC	L	-24, 28, -14	1.31(1.27)	1.21(1.43)	1.23(1.53)	1.09(1.81)	0.87(0.94)	0.278	0.672	0.247	0.091	0.377
		R	26, 24, -22	2.43(1.78)	2.83(1.85)	3.05(2.04)	2.86(2.04)	1.84(1.25)	0.181	0.191	0.008	0.175	0.406
	dlPFC	L	-42, 13, 27	0.41(0.37)	0.4(0.42)	0.49(0.43)	0.34(0.41)	0.27(0.28)	0.253	0.804	0.129	0.569	0.851
		R	48, 17, 29	0.92(1.18)	0.89(0.84)	1.03(0.92)	0.71(0.67)	0.67(0.4)	0.952	0.388	0.338	0.908	0.647
	vlPFC	L	-42, 25, 3	0.39(0.42)	0.45(0.57)	0.53(0.62)	0.54(0.77)	0.37(0.42)	0.229	0.248	0.588	0.292	0.394
		R	42, 25, 3	0.41(0.38)	0.51(0.45)	0.57(0.51)	0.45(0.62)	0.49(0.36)	0.052	0.949	0.853	0.067	0.257
	rACC	R	4, 47, 7	0.45(0.58)	0.56(0.57)	0.57(0.58)	0.45(0.68)	0.61(0.51)	0.358	0.758	0.573	0.794	0.447
	dmPFC	R	10, 54, 18	0.43(0.43)	0.51(0.41)	0.55(0.44)	0.56(0.53)	0.52(0.33)	0.369	0.649	0.492	0.521	0.453

* Due to comorbidity, some individuals are represented in more than one anxious sub-group.

All models account for (1) scanner, (2) sex, (3) race, (4) age at scan, (5) IQ, and (6) current emotional symptom scale scores. Comorbidity models include all anxiety disorders and current symptoms entered into the same model. Key: Current = current symptoms; Anx = Anxiety; GAD = Generalized Anxiety Disorder (N = 15); SAD = Separation Anxiety Disorder (N = 10); SoPh = Social Phobia (N = 11); vmPFC = Ventromedial Prefrontal Cortex; OFC = Orbital Frontal Cortex; rACC = Rostral Anterior Cingulate Cortex; dmPFC = Dorsomedial Prefrontal Cortex.

In the “comorbidity model,” which included each preschool anxiety diagnosis and school-age emotional symptom score, preschool social phobia and separation anxiety predicted different activation in both the dorsolateral and the dorsomedial prefrontal cortices in response to angry faces. In the dorsolateral prefrontal cortex, both preschool social phobia (left hemisphere; F(1,35) = 9.89, p = 0.003) and preschool separation anxiety (right hemisphere; F(1,35) = 6.50, p = 0.015) predicted less activation as compared to the non-anxious group. Both separation anxiety and social phobia also predicted differences in dorsomedial prefrontal cortex activation to angry faces, with less activation in separation anxiety (F(1,35) = 4.83, p = 0.035) and greater activation in social phobia (F(1,35) = 12.74, p = 0.001). In response to fearful faces, preschool generalized anxiety predicted greater activation in the left amygdala (F(1,35) = 4.83, p = 0.035) at school-age as compared to the non-anxious group, while controlling for other preschool anxiety disorders and school-age symptomatology.

All three anxiety disorders also predicted significantly different ventromedial prefrontal cortex activation to fearful faces. However, whereas both preschool generalized anxiety (F(1,35) = 6.29, p = 0.017) and separation anxiety (F(1,35) = 5.02, p = 0.032) predicted less activation, preschool social phobia predicted greater activation (F(1,35) = 4.53, p = 0.040). Finally, in the right lateral orbitofrontal cortex, preschool social phobia predicted less activation (F(1,35) = 7.99, p = 0.008) to fearful faces.

### Functional Connectivity (PPI) Analysis

**Figure 2:**
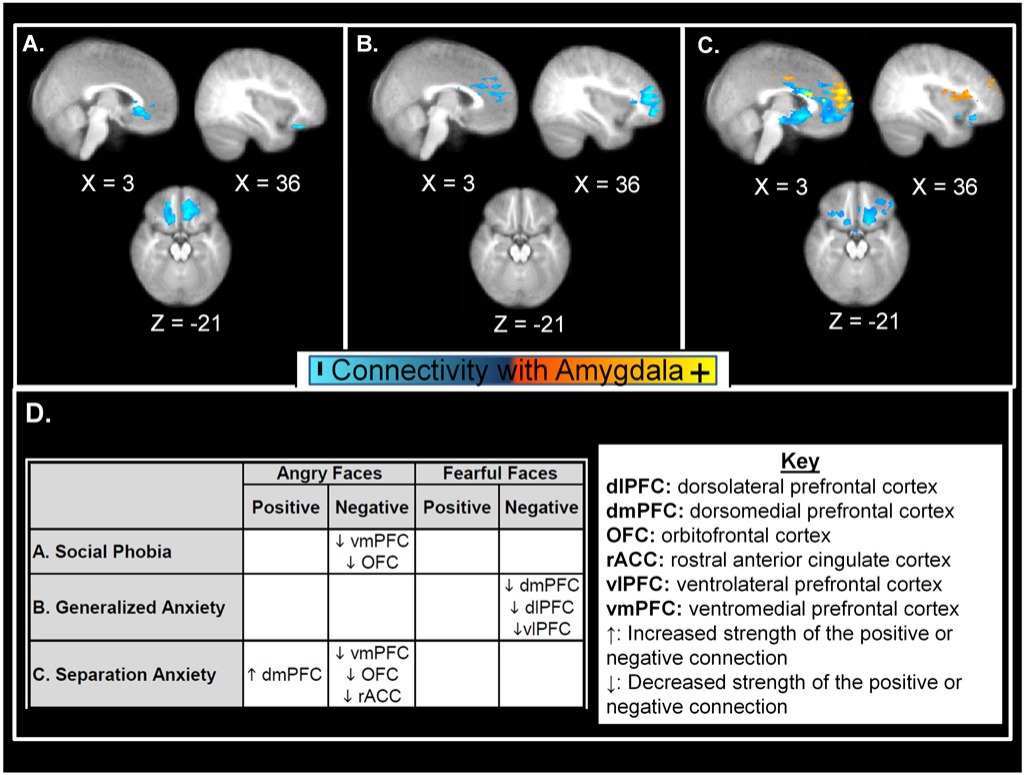
Amygdala-prefrontal connectivity in school-age children who met criteria for preschooler anxiety compared to non-anxious children. All groups were included in the model, thus these images depict the unique contribution of each disorder controlling for comorbidity with the other disorders. **(A)** Ventral prefrontal regions, including the ventromedial prefrontal cortex and orbital frontal cortex, displaying less negative connectivity with the amygdala to angry faces in social phobia (N = 11). **(B)** Dorsal prefrontal regions, including the dorsomedial, dorsolateral, and ventrolateral prefrontal cortex, displaying less negative connectivity with the amygdala to fearful faces in generalized anxiety disorder (N = 15). **(C)** Ventral prefrontal regions, including the ventromedial and orbital frontal cortices, depicting less negative connectivity (blue) with the amygdala and dorsomedial prefrontal regions depicting greater positive connectivity (yellow) with the amygdala to angry faces in separation anxiety disorder (N = 10). **(D)** Summary of PPI connectivity pattern, with an up-facing arrow denoting regions where there is an increase in the strength of the positive or negative connection in the anxiety subgroup, as compared to non-anxious children, and regions where there is a decrease in the strength of the positive or negative connection in the anxiety subgroup denoted with a down-facing arrow.

PPI results are illustrated in Fig. 2 and selected results are presented in Tables 3–5. Full results are included in S2 and S3 Tables. When controlling for comorbidity between disorders, each of the three anxiety disorders predicted different patterns of amygdala-prefrontal connectivity. Compared to children without a preschool anxiety disorder, children with preschool social phobia (Table 3; Fig. 2A) showed less school-age negative connectivity between the bilateral amygdalae (Left: z_33_=5.35, p = 0.045; Right: z_33_ = 4.11, p = 0.034) and both the right lateral orbitofrontal and the ventromedial prefrontal cortices to angry faces, but not fearful faces. Thus, in non-anxious children, there was an inverse relationship between activity in the amygdala and the prefrontal cortex to fearful faces and the strength of this correlation was decreased in children with a history of social phobia. Preschool generalized anxiety (Table 4; Fig. 2B) predicted less school-age negative connectivity between the left amygdala (z_37_ = 3.81, p = 0.002) and both the dorsomedial prefrontal and the ventrolateral/dorsolateral prefrontal cortices in response to fearful, but not angry faces. Similar to social phobia, preschool separation anxiety predicted less school-age negative connectivity to angry faces as compared to non-anxious children (Table 3; Fig. 2C). This less negative connectivity was between each amygdala (Left: z_32_=4.62, p<0.001; Right: z_32_=4.6, p<0.001) and the anterior cingulate, lateral orbitofrontal, and ventromedial prefrontal cortices. Unlike the other anxious groups, preschool separation anxiety also predicted greater *positive* connectivity between each amygdala (Left: z_32_=4.68, p<0.001; Right z_32_=5.43, p<0.001) and both the dorsolateral and the dorsomedial prefrontal cortices to angry faces at school-age (Table 5).

**Table 3:** Selected Clusters from Psychophysiological Interaction Analysis of Negative Connectivity to Angry Faces.

	**Seed Region**	**Main Cluster**		**BA**	**X**	**Y**	**Z**	Max Z*	**Size**	**Corrected p-value**
		***Local Maxima***								
**Social Phobia**	Left Amygdala	Temporal Pole/Amygdala	R	28	27	4	-26	5.35	15048	0.0453
		*Orbital Frontal*	R		22	21	-24	4.02		
		*Frontal Medial*	L		-4	39	-19	3.64		
		*Orbital Frontal*	R	47	36	35	-23	3.64		
		*Orbital Frontal*	R	47	34	34	-23	3.64		
		*Frontal Pole*	R		26	36	-23	3.6		
	Right Amygdala	Subcallosal	L		-9	29	-20	4.11	15562	0.0342
		*Orbital Frontal*	R	47	37	34	-23	3.88		
		*Frontal Pole*	R		25	37	-23	3.87		
		*Subcallosal*	R		4	28	-5	3.83		
		*Subcallosal*	L		-6	31	-23	3.79		
		*Frontal Medial*	L		-10	34	-21	3.61		
**Separation Anxiety Disorder**	Left Amygdala	Anterior Cingulate, rostral	L	24	-7	37	10	4.62	51080	6.62E-06
		*Anterior Cingulate, rostral*	L		-9	36	9	4.56		
		*Anterior Cingulate, dorsal*	R		4	22	18	4.44		
		*Anterior Cingulate, rostral*	L		-10	38	3	4.39		
		*Anterior Cingulate, rostral*	L		-7	40	4	4.36		
		*Anterior Cingulate, dorsal*	R	24	5	19	20	4.23		
	Right Amygdala	Anterior Cingulate, dorsal	R		5	20	18	4.6	90284	2.67E-09
		*Caudate*	R		14	15	10	4.29		
		*Frontal Medial*	R		9	37	-9	4.19		
		*Anterior Cingulate, rostral*	L		-9	37	7	4.18		
		*Insular*	L		-28	22	-3	4.16		
		*Subcallosal*	R	25	5	12	-11	4.16		

*Positive Z-scores represent regions where the Non-anxious group showed stronger connectivity than the Separation Anxiety Disorder or Social Phobia groups. Full tables of results can be found in supplementary materials.

**Table 4:** Selected Clusters from Psychophysiological Interaction Analysis of Negative Connectivity to Fearful Faces.

	**Seed Region**	**Main Cluster**		**BA**	**X**	**Y**	**Z**	Max Z*	**Size**	**Corrected p-value**
		***Local Maxima***								
**Generalized Anxiety Disorder**	Left Amygdala	Frontal Pole	R		27	55	0	3.81	27106	0.00153
		*Frontal Pole*	R		33	53	19	3.74		
		*Frontal Pole*	R	10	27	57	20	3.72		
		*Frontal Pole*	R		24	55	11	3.67		
		*Frontal Pole*	R		30	53	11	3.63		
		*Frontal Pole*	R		26	54	2	3.62		

*Positive Z-scores represent regions where the Non-anxious group showed stronger connectivity than the Generalized Anxiety Disorder group. Full tables of results can be found in supplementary materials.

**Table 5:** Selected Clusters from Psychophysiological Interaction Analysis of Positive Connectivity to Angry Faces.

	**Seed Region**	**Main Cluster**		**BA**	**X**	**Y**	**Z**	Max Z*	**Size**	**Corrected p-value**
		***Local Maxima***								
**Separation Anxiety Disorder**	Left Amygdala	Frontal Pole	R		9	60	19	-4.68	85995	9.08E-09
		*Frontal Pole*	R		14	63	20	-4.67		
		*Anterior Cingulate, rostral*	L	24	-7	37	10	-4.62		
		*Frontal Pole*	R		13	57	19	-4.56		
		*Anterior Cingulate, rostral*	L		-9	36	9	-4.56		
		*Anterior Cingulate, dorsal*	R		4	22	18	-4.44		
	Right Amygdala	Frontal Pole	R		15	58	21	-5.43	123648	1.05E-11
		*Frontal Pole*	R		9	59	22	-5.05		
		*Frontal Pole*	R		7	61	15	-4.89		
		*Frontal Pole*	R		18	48	22	-4.75		
		*Frontal Pole*	R		21	44	22	-4.75		
		*Frontal Pole*	L		-26	63	15	-4.71		

*Negative Z-scores represent regions where the Non-anxious group showed weaker connectivity than the Separation Anxiety Disorder group. Full tables of results can be found in supplementary materials.

As highlighted in Table 1, there were 3 non-anxious children and 13 anxious children who met criteria for a non-anxiety psychiatric disorder, including depression, ADHD, oppositional defiant disorder, or conduct disorder, during preschool. In order to ensure that our findings were not accounted for by these other comorbid disorders, we reran our analyses controlling for other disorders. Our findings remained unchanged. Compared to children without a preschool anxiety disorder, children with preschool social phobia still showed less school-age negative connectivity between the bilateral amygdalae (Left: z_33_ = 5.33, p = 0.046; Right: z_33_=3.92, p = 0.048) and both the right lateral orbitofrontal and the ventromedial prefrontal cortices to angry faces, but not fearful faces. Preschool generalized anxiety still predicted less school-age negative connectivity between the left amygdala (z_37_ = 4.01, p<0.001) and both the dorsomedial prefrontal and the ventrolateral/dorsolateral prefrontal cortices in response to fearful, but not angry faces. Finally, as compared to non-anxious children, preschool separation anxiety predicted less school-age negative connectivity between each amygdala (Left: z_32_ = 4.55, p<0.001; Right: z_32_ = 4.58, p<0.001) and the anterior cingulate, lateral orbitofrontal, and ventromedial prefrontal cortices. Preschool separation anxiety also predicted greater positive connectivity, compared to non-anxious children, between each amygdala (Left: z_32_ = 4.57, p<0.001.; Right z_32_ = 5.47, p<0.001) and both the dorsolateral and the dorsomedial prefrontal cortices to angry faces at school-age.

## Discussion

In this study we explored whether preschool generalized anxiety, separation anxiety, and/or social phobia were associated with amygdala-prefrontal dysregulation at school age. As a secondary, exploratory goal, we explored whether differences in patterns of amygdala-prefrontal dysregulation underlie phenotypic differences between specific anxiety disorders. We found that anxiety in the preschool period predicted neurobiological differences at school-age, even when accounting for concurrent school-age emotional symptoms. We also found preliminary evidence suggesting that preschool anxiety disorders are differentiated at the level of brain function based on both the stimuli that elicit the brain response and the dorsal-ventral distribution of the prefrontal regions atypically connected to the amygdala. Together, these data provide preliminary evidence that the early onset of impairing anxiety disorders may impact brain functioning in later childhood and that there may be a neurobiological basis for phenotypic differences between specific anxiety disorders.

Our findings support previous studies in older children that have shown abnormal functioning of the amygdala-prefrontal emotion regulation networks in anxiety [29–31]. In our “simple model” which did not account for comorbidity between anxiety disorders, meeting criteria for any anxiety disorder as a preschooler predicted school-age functional activation differences in one region: the dorsolateral prefrontal cortex, a region where dysregulation has been implicated in the cognitive regulation of emotional responses [37] which is a shared characteristic of all anxiety disorders. A similar pattern has been demonstrated for preschool depression, which is often comorbid with anxiety disorders and shares features with generalized anxiety [15, 20, 79]. Barch and colleagues have reported that a history of preschool onset depression predicts differences in amygdala and prefrontal responses to emotional faces that can be measured at school-age and which persist despite controlling for concurrent emotional symptoms [80]. As in our study where not all children who met criteria for a preschool anxiety disorder still met criteria at school age, not all children who met criteria for depression as preschoolers still met criteria for the disorder at school-age in their study. Together our results suggest that a history of either anxiety or depression in the preschool period is associated with neurodevelopmental processes that start early in life, specifically in amygdala and prefrontal circuitry, and that persist into school-age even if some children no longer meet criteria for the disorder.

In addition to providing evidence for the presence of enduring neural effects associated with impairing preschool anxiety disorders, our ROI analyses provide preliminary evidence for neurobiological differences between generalized anxiety, separation anxiety, and social phobia in early childhood. For example, in our “comorbidity model” we found that the decreased dorsolateral prefrontal cortex activation reported for the “all-anxious” group only remained in the subset of children with social phobia, suggesting that atypical dorsolateral prefrontal cortex development may specifically reflect early childhood social phobia, not anxious distress in general. The different activation patterns that emerged when we controlled for comorbidity between disorders provide preliminary evidence that unmodeled between-anxiety comorbidity may mask interesting, disorder-specific differences.

In addition to regional activation differences for the specific anxiety disorders when we controlled for comorbidity between disorders, we found preliminary evidence for anxiety disorder-specific differences in (1) the pattern of amygdala-prefrontal connectivity and (2) the negative face condition (i.e., angry or fearful faces) that elicited the difference in connectivity response. Previous work suggests that fearful and angry faces depict different types of information, with angry faces imparting information about a directed social threat and fearful faces imparting information about ambiguous threats in the environment [81]. In our study, fearful and angry faces elicit disorder-specific patterns of amygdala-prefrontal connectivity. Differences in the pattern of disruptions in amygdala-prefrontal connectivity may result in disruptions in different aspects of emotion regulation, resulting in different patterns of anxious distress that are reflected in the unique phenotypic expression associated with our anxiety disorder sub-types.

Our data suggests that early childhood generalized anxiety may reflect disruption of effortful emotion regulation processes, such as suppression and reappraisal, involve regulation of the amygdala by the dorsal prefrontal cortices [37–39]. We found that a history of preschool generalized anxiety disorder predicted decreased school-age negative connectivity between the amygdala and both the dorsomedial and ventrolateral/dorsolateral prefrontal cortices in response to fearful face, but not to angry faces. These amygdala-dorsal prefrontal systems have been implicated in worry [35, 36], and disrupted amygdala-dorsal prefrontal connectivity has been found in adults with generalized anxiety disorder [82, 83], suggesting that dysregulation between the amygdala and the dorsal prefrontal cortices underlies the worries and rumination that characterize generalized anxiety. The atypical connectivity between the amygdala and dorsal prefrontal networks associated with generalized anxiety disorder may contribute to the increased amygdala activation to negative faces reported previously [49, 50, 84] and replicated in our ROI analyses. Our data suggest that dysregulation of this amygdala-dorsal prefrontal network and generalized anxiety symptoms can emerge early in development.

Our data suggest that early childhood social phobia may reflect dysregulation of automatic emotional regulation and fear extinction processes that result in the hypervigilance to social information and the inability to regulate the resulting fear response that is characteristic of this disorder. In our study, preschool social phobia predicted less school-age negative connectivity between the amygdala and a ventral prefrontal network, including the lateral orbitofrontal cortex, ventromedial prefrontal cortex, and anterior cingulate cortex, which previous studies have shown are associated with automatic emotion regulation and fear extinction processes [37–39]. Our finding in young children replicates previous reports of less amygdala-ventral prefrontal cortex connectivity in adults with social phobia [85–87], and suggests that atypical development of this network emerges early in development.

Our data suggest that early childhood separation anxiety may reflect HPA axis dysregulation. We found that a history of preschool separation anxiety predicted a unique pattern of amygdala-prefrontal connectivity, with less negative amygdala-ventromedial prefrontal cortex connectivity and greater *positive* amygdala-dorsomedial prefrontal cortex (BA10/46) connectivity to angry faces at school-age. The dorsal prefrontal cortex imparts its influence on the amygdala via shared connections with more ventral portions of the prefrontal cortex [88, 89]. Greater activation in the dorsomedial prefrontal cortex and negative correlations between the ventromedial prefrontal cortex and the amygdala are associated with better HPA axis regulation [90, 91]. Our results, while preliminary, provide some evidence suggesting that dysregulation of the pathway from the dorsomedial to the ventromedial prefrontal cortex and the associated inhibition of the amygdala response may underlie previous reports of HPA-axis dysregulation in separation anxiety [51]. This interpretation is consistent with non-human primate studies of the neural circuits activated by separation distress [52] and thus provides a possible neurobiological mechanism for the developmentally inappropriate and excessive separation distress characteristic of this disorder.

Both preschool separation anxiety and social phobia predicted different school-age connectivity in response to angry faces, but not fearful faces as found with preschool generalized anxiety. These anxiety phenotype-specific atypical activation responses to different types of negative emotional stimuli suggest that children with early generalized anxiety respond atypically to ambiguous environmental threats (e.g. fearful faces) whereas children with separation anxiety and social phobia respond atypically to direct social threats (e.g. angry faces). These differences in neural connectivity by stimulus type are consistent with the phenotypic differences between the social fears and avoidance that characterize separation anxiety and social phobia and the ruminative worries characteristic of generalized anxiety.

Although our finding of decreased negative amygdala-prefrontal connectivity in generalized anxiety and social phobia replicate several previous studies (there are no imaging studies of separation anxiety), other groups report increased positive amygdala-prefrontal connectivity in these disorders [82, 92, 93]. Our data suggest that decreased negative connectivity is not associated with a concomitant increase in positive connectivity, as there were no networks in which children with a history of either preschool generalized anxiety or social phobia displayed increased positive connectivity as compared to non-anxious children (see S3 Table). One explanation for these different findings is our use of the star stimuli as the baseline condition. Many previous studies have used neutral faces as a baseline condition, which controls for emotion-general effects, but may result in unintended bias based on evidence that neutral faces are not processed the same in social phobia as they are in unaffected individuals [62]. A more compelling explanation for these differences is that many of the previous studies ignored the influence of between-anxiety comorbidity, which, as shown in our ROI analyses, may mask important differences between the disorders. Because of our modest sample size and high levels of comorbidity between anxiety disorders, we were underpowered to test whole-brain differences between specific anxiety disorders. Thus, it was impossible to test whether the patterns of amygdala-prefrontal dysregulation between each disorder and the non-anxious children was also different between anxiety disorders and therefore our specificity results represent exploratory, preliminary findings needing replication.

Our study has a number of limitations. Although our sample is drawn from a larger community pediatric primary care study, not a clinical sample, the sample size for this first wave of our imaging study is modest, although similar to other neuroimaging studies in young children [80]. Our relatively small sample size reflects the challenge of conducting fMRIs with children under the age of seven, many of whom are anxious. We achieved a significantly better success rate of 76% at our second functional MRI time point, where we scanned older children (ages 6–10 years old) on an almost identical paradigm to the one used in the present paper. One solution for increasing our power would have been to use continuous symptom counts, as opposed to the dichotomous diagnostic variables, in our analyses. We chose against this option because the number of symptoms comprising each anxiety sub-type is variable, with social phobia in particular being comprised of fewer symptoms. This would have introduced an undesirable bias in our analyses. Another limitation is that we did not have the power to examine differences between children who “outgrow” their preschool anxiety diagnosis and those who maintain or develop anxiety in later childhood. Finally, without concurrent eye-tracking during the scans, it is impossible to ensure that the participants were fully engaged with the stimuli throughout the scan. In an effort to keep the children engaged, we asked them to press a button to faces wearing glasses. While this helped ensure that the children were attending to the stimuli, it necessarily drew the children’s attention to the eye area of the face. Previous eye-tracking studies demonstrate that children with anxiety display different attentional bias to emotional faces (reviewed in [94]). Future studies using concurrent eye-tracking are needed to determine whether differences in attention to the eye area of the faces drove our imaging findings. While our study provides the first glimpse into the neurobiological correlates of preschool anxiety disorders, the results must be interpreted with caution in light of these limitations until they are replicated in a larger cohort of children.

In summary, we found support for our hypothesis that impairing anxiety in the preschool period is associated with neural effects at school age that occur over-and-above the effects of school-age emotional symptoms. We also found preliminary support for our exploratory hypothesis that phenotypic differences between anxiety disorders may be associated with disorder-specific regulation of amygdala-prefrontal networks. By understanding the neurobiological mechanisms that contribute to the development of, and the phenotypic distinction between, impairing anxiety disorders in early childhood, we may be able eventually to identify biomarkers for early risk and early symptoms, and to develop measurable, disorder-specific targets for intervention. Early identification and intervention may enable us to decrease distress and impairment in young children with clinically significant anxiety, to alter children’s neurodevelopmental trajectory during a period when they are developing the neurobiological pathways supporting emotion regulation skills [27] and thus to decrease young children’s risk for impairing emotional disorders in adolescence and adulthood.

## Supporting Information

S1 Fig Location of each of the region of interests. ROIs consisted of 6mm spheres formed around coordinates from meta-analyses of the neural bases of face-processing, emotion perception, emotion regulation, and threat appraisal [72–75].
(TIF)

S2 Fig Activation in the entire sample of children (N = 45) in response to face stimuli. Significant activation clusters were identified in the face-processing network, including the (A) amygdala, as well as the (B) fusiform gyrus and prefrontal cortex. For reference, panel (C) includes the posterior probability map produced from a query of “faces” in the NeuroSynth meta-analysis atlas [95].
(TIF)

S3 Fig Graphs of significant ROI findings. All data points represent model corrected predictive values. *Note: some individuals are represented in more than one anxiety group, namely generalized anxiety disorder (GAD), separation anxiety disorder (SAD), or social phobia (SoPh).
(TIF)

S1 Table Complete region of interest results. (PDF)

S2 Table Negative Connectivity with the Amygdala. (PDF)

S3 Table Positive Connectivity with the Amygdala. (PDF)

S1 Text Supplementary Methods. (DOCX)
