# Supplementary figures and images for: Preschool Anxiety Disorders Predict Different Patterns of Amygdala-Prefrontal Connectivity at School-Age
Source: PLoS One. 2015 Jan 27;10(1):e0116854. doi: 10.1371/journal.pone.0116854 (PMC4308069; doi:10.1371/journal.pone.0116854)

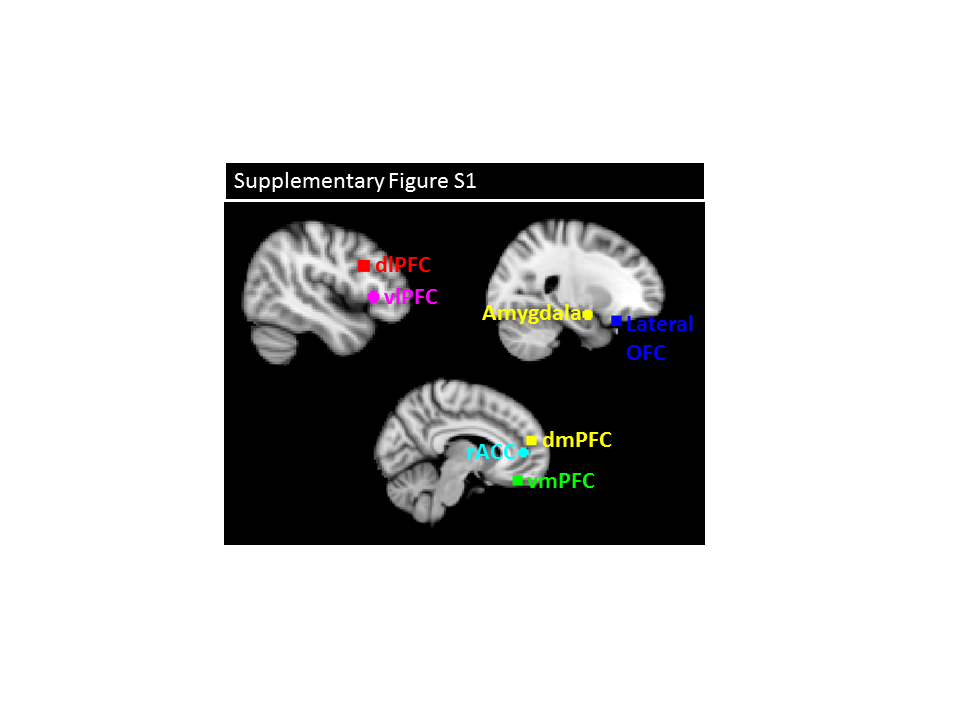

Supplement: S1 Fig — ROIs consisted of 6mm spheres formed around coordinates from meta-analyses of the neural bases of face-processing, emotion perception, emotion regulation, and threat appraisal [72–75]. (TIF) [file pone.0116854.s001.tif]

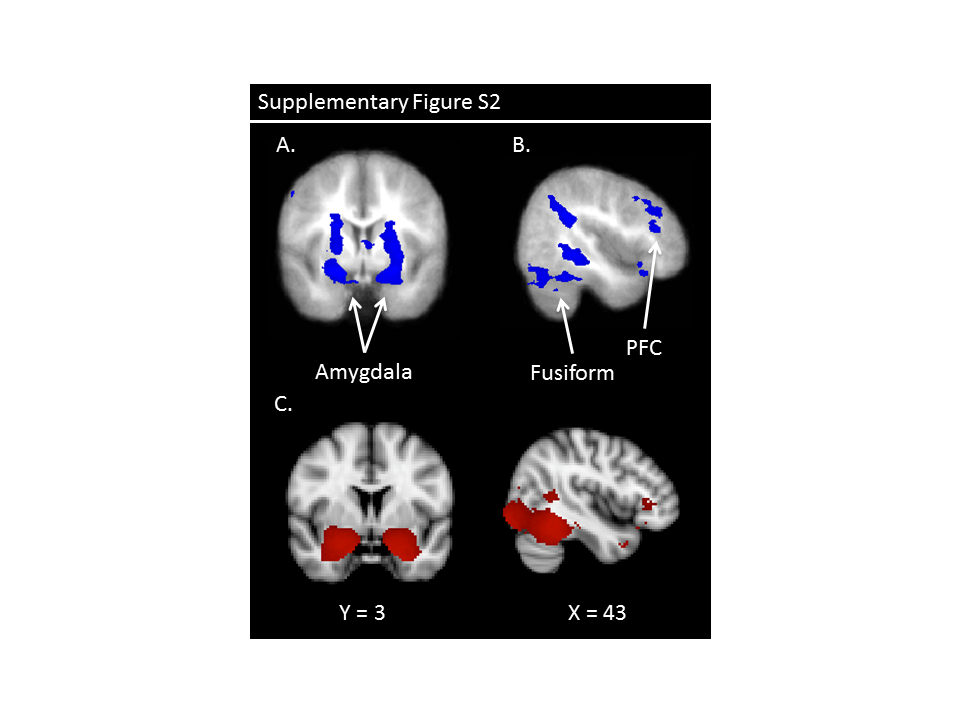

Supplement: S2 Fig — Significant activation clusters were identified in the face-processing network, including the (A) amygdala, as well as the (B) fusiform gyrus and prefrontal cortex. For reference, panel (C) includes the posterior probability map produced from a query of “faces” in the NeuroSynth meta-analysis atlas [95]. (TIF) [file pone.0116854.s002.tif]

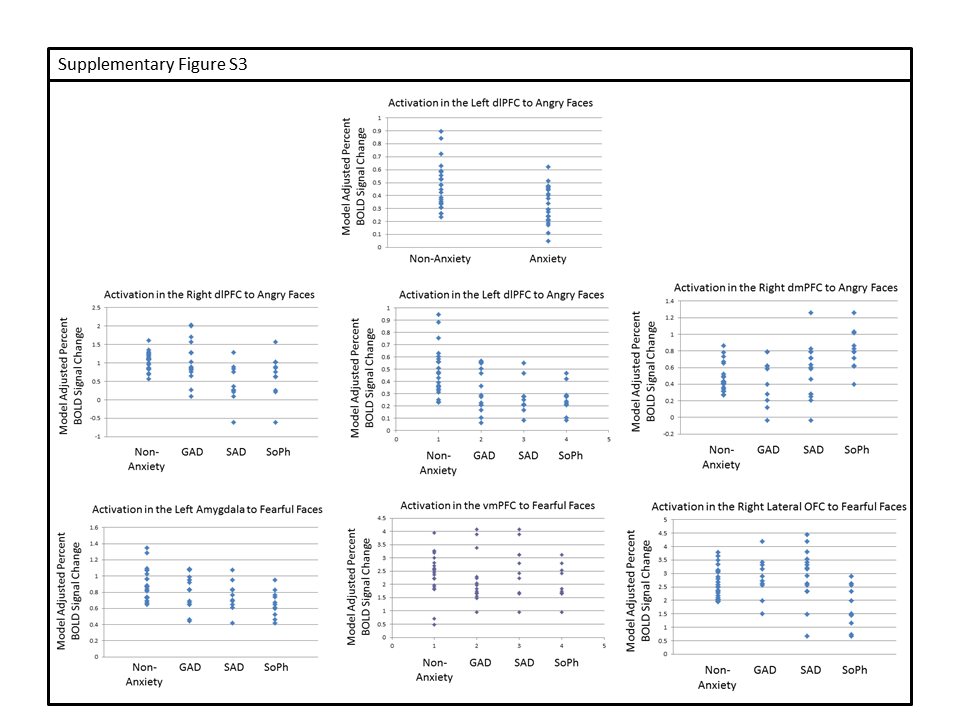

Supplement: S3 Fig — All data points represent model corrected predictive values. *Note: some individuals are represented in more than one anxiety group, namely generalized anxiety disorder (GAD), separation anxiety disorder (SAD), or social phobia (SoPh). (TIF) [file pone.0116854.s003.tif]
