# Supplementary material for: Preschool Anxiety Disorders Predict Different Patterns of Amygdala-Prefrontal Connectivity at School-Age
Source: PLoS One. 2015 Jan 27;10(1):e0116854. doi: 10.1371/journal.pone.0116854 (PMC4308069; doi:10.1371/journal.pone.0116854)
Supplement: S1 Table — (PDF) [file pone.0116854.s004.pdf]

Supplementary Table S1. Complete region of interest results

Regions		MNI Coordinates	Max Percent Signal Change - Unadjusted Mean (standard deviation) (1) Non-Anxious N=23	(2) Any Anxiety N=22	(3) GAD N=15*	(4) SAD N=10*	(5) SOC N=11*	Adjusted p-values Simple Model 1 vs. 2	1 vs. 3	1 vs. 4	1 vs. 5
**Angry Faces**											
Amygdala[1]	L	-20, -6, -15	0.88 (0.68)	0.80 (0.52)	0.85 (0.52)	0.80 (0.65)	0.69 (0.42)	0.398	0.425	0.527	0.200
	R	20, -4, -15	1.12 (0.77)	0.90 (0.72)	0.93 (0.70)	0.95 (0.82)	0.98 (0.77)	0.147	0.160	0.476	0.338
vmPFC[3]	M	0, 38, -18	2.46 (1.86)	2.01 (1.87)	2.03 (1.78)	2.22 (2.34)	1.85 (1.92)	0.688	0.692	0.993	0.503
	R	6, 40, -22	3.18 (2.71)	3.06 (2.87)	3.17 (2.92)	2.93 (2.85)	2.54 (2.99)	0.522	0.709	0.449	0.417
Lateral OFC[2]	L	-24, 28, -14	1.18 (1.10)	1.04 (0.86)	1.11 (0.82)	0.72 (0.66)	0.75 (0.81)	0.787	0.875	0.212	0.244
	R	26, 24, -22	2.70 (2.03)	2.76 (2.24)	2.98 (2.32)	3.05 (2.49)	2.04 (1.88)	0.900	0.884	0.958	0.261
dlPFC[1]	L	-42, 13, 27	0.47 (0.39)	0.34 (0.27)	0.36 (0.29)	0.30 (0.28)	0.28 (0.25)	0.041	0.085	0.057	0.001
	R	48, 17, 29	0.99 (0.93)	0.95 (1.30)	1.11 (1.50)	0.52 (0.48)	0.65 (0.45)	0.755	0.981	0.115	0.134
vlPFC[1]	L	-42, 25, 3	0.62 (0.53)	0.51 (0.51)	0.54 (0.58)	0.67 (0.69)	0.43 (0.35)	0.827	0.921	0.439	0.038
	R	42, 25, 3	0.56 (0.43)	0.51 (0.36)	0.50 (0.41)	0.44 (0.46)	0.48 (0.25)	0.499	0.585	0.353	0.008
rACC[1]	R	4, 47, 7	0.48 (0.56)	0.46 (0.55)	0.39 (0.53)	0.36 (0.58)	0.56 (0.55)	0.727	0.595	0.725	0.621
dmPFC[4]	R	10, 54, 18	0.48 (0.36)	0.57 (0.57)	0.53 (0.56)	0.47 (0.65)	0.82 (0.53)	0.586	0.362	0.390	0.001
**Fearful Faces**											
Amygdala[1]	L	-20, -6, -15	0.70 (0.60)	0.87 (0.77)	1.03 (0.85)	0.89 (0.96)	0.68 (0.53)	0.135	0.071	0.243	0.845
	R	20, -4, -15	0.96 (0.70)	1.00 (0.74)	1.13 (0.76)	1.16 (0.96)	1.11 (0.78)	0.613	0.412	0.307	0.964
vmPFC[3]	M	0, 38, -18	2.28 (1.90)	1.54 (1.40)	1.21 (1.01)	1.34 (1.13)	2.14 (1.60)	0.228	0.022	0.066	0.924
	R	6, 40, -22	3.42 (2.90)	2.29 (2.00)	2.14 (2.09)	2.17 (2.39)	2.72 (1.84)	0.309	0.358	0.237	0.204
Lateral OFC[2]	L	-24, 28, -14	1.31 (1.27)	1.21 (1.43)	1.23 (1.53)	1.09 (1.81)	0.87 (0.94)	0.377	0.350	0.580	0.406
	R	26, 24, -22	2.43 (1.78)	2.83 (1.85)	3.05 (2.04)	2.86 (2.04)	1.84 (1.25)	0.406	0.283	0.435	0.402
dlPFC[1]	L	-42, 13, 27	0.41 (0.37)	0.40 (0.42)	0.49 (0.43)	0.34 (0.41)	0.27 (0.28)	0.851	0.549	0.716	0.145
	R	48, 17, 29	0.92 (1.18)	0.89 (0.84)	1.03 (0.92)	0.71 (0.67)	0.67 (0.40)	0.647	0.731	0.458	0.172
vlPFC[1]	L	-42, 25, 3	0.39 (0.42)	0.45 (0.57)	0.53 (0.62)	0.54 (0.77)	0.37 (0.42)	0.394	0.330	0.267	0.860
	R	42, 25, 3	0.41 (0.38)	0.51 (0.45)	0.57 (0.51)	0.45 (0.62)	0.49 (0.36)	0.257	0.130	0.546	0.989
rACC[1]	R	4, 47, 7	0.45 (0.58)	0.56 (0.57)	0.57 (0.58)	0.45 (0.68)	0.61 (0.51)	0.447	0.384	0.801	0.761
dmPFC[4]	R	10, 54, 18	0.43 (0.43)	0.51 (0.41)	0.55 (0.44)	0.56 (0.53)	0.52 (0.33)	0.453	0.345	0.369	0.581

References

1. Sabatinelli D, Fortune EE, Li Q, Siddiqui A, Krafft C, Oliver WT, et al. Emotional perception: meta-analyses of face and natural scene processing. Neuroimage. 2011;54(3):2524-33.

2. Banks SJ, Eddy KT, Angstadt M, Nathan PJ, Phan KL. Amygdala-frontal connectivity during emotion regulation. Soc Cogn Affect Neurosci. 2007;2(4):303-12.

3. Diekhof EK, Geier K, Falkai P, Gruber O. Fear is only as deep as the mind allows: a coordinate-based meta-analysis of neuroimaging studies on the regulation of negative affect. Neuroimage. 2011;58(1):275-85.

4. Thayer JF, Ahs F, Fredrikson M, Sollers JJ, 3rd, Wager TD. A meta-analysis of heart rate variability and neuroimaging studies: implications for heart rate variability as a marker of stress and health. Neuroscience and biobehavioral reviews. 2012;36(2):747-56

* Due to comorbidity, some individuals are represented in more than one anxious sub-group

All models account for (1) scanner, (2) sex, (3) race, (4) age at scan, (5) IQ, and (6) current emotional symptom scale score.

Key: GAD = Generalized Anxiety Disorder; SAD = Separation Anxiety Disorder; SoPh = Social Phobia; vmPFC = ventromedial Prefrontal Cortex; OFC =
