# Supplementary material for: Preschool Anxiety Disorders Predict Different Patterns of Amygdala-Prefrontal Connectivity at School-Age
Source: PLoS One. 2015 Jan 27;10(1):e0116854. doi: 10.1371/journal.pone.0116854 (PMC4308069; doi:10.1371/journal.pone.0116854)
Supplement: S2 Table — (PDF) [file pone.0116854.s005.pdf]

Supplementary Table S2. Negative Connectivity with the Amygdala

Main Cluster / Local Maxima		BA	X	Y	Z	Max Z	Size	Corrected p-value
**Clusters negatively connected with the left amygdala to angry faces**								
**Non-Anxious**								
Orbital Frontal Cortex	R	47	25	21	-26	5	140778	1.45E-12
Orbital Frontal Cortex	R		23	20	-24	4.88		
Orbital Frontal Cortex	R	1	28	26	-24	4.68		
Orbital Frontal Cortex	R	11	30	27	-24	4.59		
Frontal Medial Cortex	L		-5	39	-19	4.5		
Brainstem	L		-2	-12	-17	4.5		
**GAD**								
None								
**SAD**								
None								
**SoPh**								
None								
**Non-Anxious > SAD**								
Anterior Cingulate Gyrus, rostral	L	24	-7	37	10	4.62	51080	6.62E-06
Anterior Cingulate Gyrus, rostral	L		-9	36	9	4.56		
Anterior Cingulate Gyrus, dorsal	R		4	22	18	4.44		
Anterior Cingulate Gyrus, rostral	L		-10	38	3	4.39		
Anterior Cingulate Gyrus, rostral	L		-7	40	4	4.36		
Anterior Cingulate Gyrus, dorsal	R	24	5	19	20	4.23		
**Non-Anxious > SoPh**								
Temporal Pole/Amygdala	R	28	27	4	-26	5.35	15048	0.0453
Orbital Frontal Cortex	R		22	21	-24	4.02		
Frontal Medial Cortex	L		-4	39	-19	3.64		
Orbital Frontal Cortex	R	47	36	35	-23	3.64		
Orbital Frontal Cortex	R	47	34	34	-23	3.64		
Frontal Pole	R		26	36	-23	3.6		
**Clusters negatively connected with the right amygdala to angry faces**								
**Non-Anxious**								
Subcallosal Cortex	R	24	4	33	-10	4.55	135068	1.78E-12
Subcallosal Cortex	L		-9	29	-20	4.43		
Frontal Pole	L		-36	41	-20	4.37		
Subcallosal Cortex	R		10	30	-11	4.25		
Subcallosal Cortex	R	24	4	26	-9	4.17		
Orbital Frontal Cortex	R		11	23	-20	4.13		
**GAD**								
None								
**SAD**								
None								
**SoPh**								
Frontal Pole	R	9	9	59	30	3.89	20065	0.00892
Inferior Frontal Gyrus, pars triangu	R		53	31	11	3.58		
Inferior Frontal Gyrus, pars triangu	R		55	30	12	3.5		
Frontal Pole	L	9	-15	64	22	3.4		
Frontal Pole	R		36	54	21	3.31		
Frontal Pole	R		18	49	21	3.3		
Frontal Pole	R		13	43	45	3.28	14863	0.0425
Frontal Pole	R		19	46	39	3.28		
Frontal Pole	R		15	43	51	3.26		
Superior Frontal Gyrus	R		7	25	56	3.2		
Frontal Pole	R		18	51	39	3.16		
Superior Frontal Gyrus	L		-9	36	49	3.13		
**Clusters negatively connected with the right amygdala to angry faces**								
**SoPh>SAD**								
Frontal Pole	R		15	57	21	5.16	19174	0.0116
Frontal Pole	R		10	58	29	4.84		
Frontal Pole	R		9	59	22	4.71		
Frontal Pole	R		8	62	15	4.63		
Frontal Pole	R		14	63	14	4.58		
Frontal Pole	L		-26	63	15	4.43		
**Non-Anxious > SAD**								
Anterior Cingulate Gyrus, dorsal	R		5	20	18	4.6	90284	2.67E-09
Caudate	R		14	15	10	4.29		
Frontal Medial Cortex	R		9	37	-9	4.19		
Anterior Cingulate Gyrus, rostral	L		-9	37	7	4.18		
Insular Cortex	L		-28	22	-3	4.16		
Subcallosal Cortex	R	25	5	12	-11	4.16		
**Non-Anxious > SoPh**								
Subcallosal Cortex	L		-9	29	-20	4.11	15562	0.0342
Orbital Frontal Cortex	R	47	37	34	-23	3.88		
Frontal Pole	R		25	37	-23	3.87		
Subcallosal Cortex	R		4	28	-5	3.83		
Subcallosal Cortex	L		-6	31	-23	3.79		
Frontal Medial Cortex	L		-10	34	-21	3.61		
**Clusters negatively connected with the left amygdala to fearful faces**								
**Non-Anxious**								1.18E-20
Paracingulate Gyrus	R	9	5	53	14	4.49	282614	
Frontal Pole	R		39	39	26	4.46		
Frontal Pole	R		24	63	13	4.45		
Lateral Occipital Cortex, inferior division	L	19	-55	-69	13	4.43		
Frontal Pole	R		45	40	-10	4.41		
Frontal Pole	R		41	51	-6	4.39		
**GAD**								
None								
**SAD**								
None								
**SoPh**								0.00309
Lateral Occipital Cortex, superior division	R		12	-61	72	3.89	24424	
Lateral Occipital Cortex, superior division	R		12	-63	73	3.88		
Postcentral Gyrus	R	4	62	-16	36	3.86		
Lateral Occipital Cortex, superior division	R		7	-64	73	3.55		
Superior Temporal Gyrus, posterior division	R		66	-29	19	3.51		
Supramarginal Gyrus, anterior division	R		64	-28	33	3.35		0.0344
Frontal Pole	L		-8	58	-30	3.69	15896	
Frontal Pole	R		38	51	-19	3.33		
Frontal Pole	R		29	54	-18	3.18		
Frontal Medial Cortex	R		2	32	-27	3.17		
Subcallosal Cortex	R		4	28	-15	3.13		
Frontal Pole	R		26	56	-17	3.11		
**Non-Anxious > GAD**								0.00153
Frontal Pole	R		27	55	0	3.81	27106	
Frontal Pole	R		33	53	19	3.74		
Frontal Pole	R	10	27	57	20	3.72		
Frontal Pole	R		24	55	11	3.67		
Frontal Pole	R		30	53	11	3.63		
Frontal Pole	R		26	54	2	3.62		
