# Supplementary material for: Preschool Anxiety Disorders Predict Different Patterns of Amygdala-Prefrontal Connectivity at School-Age
Source: PLoS One. 2015 Jan 27;10(1):e0116854. doi: 10.1371/journal.pone.0116854 (PMC4308069; doi:10.1371/journal.pone.0116854)
Supplement: S3 Table — (PDF) [file pone.0116854.s006.pdf]

Supplementary Table S3. Positive Connectivity with the Amygdala

Main Cluster / Local Maxima		BA	X	Y	Z	Max Z	Size	Corrected p-value
**Clusters Positively connected with the left amygdala to angry faces**								
**Non-Anxious**								
Postcentral Gyrus	L		-62	-11	47	4.77	57579	1.79E-06
Frontal Pole	L		-41	53	35	4.74		
Parahippocampal Gyrus	L		-19	-23	-22	4.73		
Parahippocampal Gyrus	L		-19	-25	-23	4.65		
Postcentral Gyrus	L		-67	-6	36	4.5		
Precentral Gyrus	L	6	-63	2	39	4.42		
**GAD**								
None								
**SAD**								
Frontal Pole	R	9	13	57	19	4.38	144063	8.92E-13
Frontal Pole	R		14	63	20	4.35		
Posterior Cingulate Gyrus*	L		-19	-16	39	4.25		
Frontal Pole	R	9	9	59	20	4.22		
Frontal Pole	R		37	35	-9	4.14		
Frontal Pole	R		3	61	23	4.08		
**SoPh**								
None								
**SAD > SoPh**								
Frontal Pole	L		-18	71	15	4.18	39012	9.05E-05
Frontal Pole	R		18	59	30	4.14		
Frontal Pole			19	64	21	4.13		
Frontal Pole	R		13	59	20	4.01		
Frontal Pole*	L	46	-52	45	23	3.98		
Frontal Pole	L	46	-49	46	23	3.97		
Postcentral Gyrus	L		-18	-31	78	4.04	24144	0.00342
Superior Frontal Gyrus	L	6	-15	11	60	3.69		
Posterior Cingulate Gyrus*	L		-19	-16	39	3.69		
Middle Frontal Gyrus*	L	6	-27	8	40	3.57		
Posterior Cingulate Gyrus*	L	31	-24	-25	41	3.53		
Superior Frontal Gyrus	L		-21	31	49	3.53		
**SAD > Non-Anxious**								
Frontal Pole	R		9	60	19	4.68	85995	9.08E-09
Frontal Pole	R		14	63	20	4.67		
Anterior Cingulate Gyrus, rostral	L	24	-7	37	10	4.62		
Frontal Pole	R		13	57	19	4.56		
Anterior Cingulate Gyrus, rostral	L		-9	36	9	4.56		
Anterior Cingulate Gyrus, dorsal	R		4	22	18	4.44		

Main Cluster / Local Maxima		BA	X	Y	Z	Max Z	Size	Corrected p-value
**Clusters Positively connected with the right amygdala to angry faces**								
**Non-Anxious**								
None								
**GAD**								
None								
**SAD**								
Frontal Pole	R		15	58	21	5.6	207305	6.22E-17
Frontal Pole	R		9	59	22	5.25		
Frontal Pole	R		8	61	15	4.96		
Frontal Pole	R		20	48	21	4.94		
Frontal Pole	R		14	61	15	4.84		
Frontal Pole	R		10	58	29	4.81		
Middle Temporal Gyrus, temporooccipital part	L		-44	-52	2	4.03	37257	0.000105
Precuneus Cortex	R		7	-69	28	3.99		
Precuneus Cortex	R		4	-68	28	3.92		
Lateral Occipital Cortex, inferior division	L	19	-48	-74	10	3.73		
Angular Gyrus	L	39	-61	-56	32	3.66		
Lateral Occipital Cortex, superior division	L		-50	-69	16	3.62		
**SoPh**								
None								
**SAD > GAD**								
Frontal Pole	R		15	59	21	4.56	24728	0.00244
Frontal Pole	R		9	59	22	4.1		
Frontal Pole	L		-26	63	15	3.96		
Frontal Pole	R		8	64	14	3.79		
Frontal Pole	R		8	61	15	3.75		
Frontal Pole	L	10	-23	62	18	3.67		
**SAD > SoPh**								
Frontal Pole	R		15	57	21	5.16	117621	2.75E-11
Frontal Pole	R		10	58	29	4.84		
Frontal Pole	R		9	59	22	4.71		
Frontal Pole	R		8	62	15	4.63		
Frontal Pole	R		14	63	14	4.58		
Frontal Pole	L		-26	63	15	4.43		
**SAD > Non-Anxious**								
Frontal Pole	R		15	58	21	5.43	123648	1.05E-11
Frontal Pole	R		9	59	22	5.05		
Frontal Pole	R		7	61	15	4.89		
Frontal Pole	R		18	48	22	4.75		
Frontal Pole	R		21	44	22	4.75		
Frontal Pole	L		-26	63	15	4.71		
Lateral Occipital Cortex, superior division	L		-55	-67	27	3.83	20721	0.00739
Lateral Occipital Cortex, superior division	L		-49	-67	29	3.83		
Precuneus Cortex	R		1	-70	44	3.79		
Lateral Occipital Cortex, superior division	L		-46	-65	29	3.74		
Lateral Occipital Cortex, superior division	L		-44	-63	28	3.66		
Lateral Occipital Cortex, superior division	L		-49	-66	32	3.63		
