# Supplementary material for: Preschool Anxiety Disorders Predict Different Patterns of Amygdala-Prefrontal Connectivity at School-Age
Source: PLoS One. 2015 Jan 27;10(1):e0116854. doi: 10.1371/journal.pone.0116854 (PMC4308069; doi:10.1371/journal.pone.0116854)
Supplement: S1 Text — (DOCX) [file pone.0116854.s007.docx]

Supplementary Text S1: Supplementary Methods

Study Design & Participants – The Preschool Anxiety Study

Participants for the current study were recruited from a larger study of preschool anxiety (Figure 1), a screen-stratified, cross-sectional study with three phases: (i) a primary care questionnaire screening phase, (ii) an in-home parent interview phase, and (iii) a lab-based case-control phase. A summary of the study design details pertinent for the current manuscript follows, however a complete description of the Preschool Anxiety Study can be found in.^1^

The Preschool Anxiety Study sample included 3,433 children attending pediatric primary care clinics for both well-child and sick-child visits who were screened when they were aged 2 through 5 years. When the child and their caregiver were in the examination room, the nurse asked the caregiver whether they were willing to speak with the screener regarding participation in a research study. If the caregiver agreed, the screener would obtain written consent from the caregiver and administer a 10-item anxiety screening measure. This measure was developed using data from our earlier preschool study^2^ in which we screened 1,220 parents of preschoolers in pediatric primary care using the CBCL 1 ½-5^3^ and completed Preschool Age Psychiatric Assessments (PAPA) and Strength and Difficulty Questionnaires^44^ with 307 parents. Children were identified as screen positive if the parents endorsed 4 or more of the 10 items on this screener. Inclusion criteria for the screening phase of the Preschool Anxiety Study were (i) the child was between 24 and 71 months old, (ii) the child attended the pediatric clinic during a screening period. Exclusion criteria were (i) the child was not accompanied by a parent/legal guardian who could provide consent, (ii) the parent/legal guardian lacked adequate fluency in English to complete the screen, (iii) the index child was known to have mental retardation (IQ < 70), autism, or other pervasive developmental disorders, (iv) the child’s sibling was participating in the study, or (v) the provider decided that the child was too medically ill at the visit for the parent to be approached about the study.

4,520 children ages 2-5 years old attended the pediatric clinics on the screening days. 519 (11.5%) were excluded from screening, 522 (13%) parents refused to participate in screening, and we missed making contact with 46 parents (1.1%). Thus, of the 4,001 eligible children, we screened 3,433 (85.8%), of which 944 (27.5%) screened high on the anxious/depressed scale, and 2,490 (72.5%) did not screen high. All children who screened high and a random sample of 189 (7.5%) who did not screen high were selected to participate in phase 2: the in-home assessment phase. Screening took place over 45 months.

Of the 1,132 children selected to participate in the in-home assessment phase, 917 (82.4%) eligible parents completed the PAPA and checklist assessments about the child’s psychiatric symptoms and temperament, the parent’s own personality traits, symptoms of anxiety, and depression, and distress in the parent-child relationship. In-home assessments took place over 47 months.

In phase 3, the lab-based case-control phase, children were identified with the PAPA as either (i) meeting criteria for generalized anxiety disorder, separation anxiety disorder, and/or social phobia or (ii) not meeting criteria for any of these anxiety disorders. 502 children participated in the lab-based case-control phase, 254 of whom met criteria for generalized anxiety disorder, separation anxiety disorder, and/or social phobia (anxious group), and 248 who did not meet criteria for any of these anxiety disorders (non-anxious group). The lab assessment was multi-modal (questionnaires, observational and experimental measures) and included assessments of temperament characteristics (including negative affectivity, effortful control, and behavioral inhibition and disinhibition), social-competence, parent-child interactions, and parental psychiatric history. The lab-based case-control phase took place over 39 months.

Study Design & Participants – The Learning About the Developing Brain Study

The Learning About the Developing Brain study, an extension of the Preschool Anxiety Study (Figure 1), was a 5 year longitudinal study of early childhood brain development and anxiety disorders. Data was collected yearly over 5 waves of data collection. With each wave of data collection, children participated in a laboratory visit that included a multi-modal battery of questionnaires and observational measures aimed at assessing the child’s temperament characteristics (including negative affectivity and effortful control) and social-competence, as well as parent-child interactions and parental psychiatric history. Each laboratory visit also included an eye-tracking session to assess attentional processing in the children. Once a child turned 6 years old, they were eligible for a follow-up clinical assessment using the PAPA and for measurement of their intellectual functioning with the Differential Ability Scale.

452 subjects who had participated in phase 3 of the Preschool Anxiety study, the lab-based case-control phase, were eligible to participate in the Learning About the Developing Brain study. Subjects were eligible if they met the same inclusion/exclusion criteria as described for the Preschool Anxiety study and were between the ages of 4 and 8 during the first wave of data collection. Of the 50 ineligible children, 38 were too young to participate, 2 were too old, 6 children had moved out of state, 2 had habitually failed to come to appointments during the Preschool Anxiety study, 1 was excluded because of a physical disability which kept the child from being able to perform the eye-tracking component of the study, and 1 was excluded due to IQ below 70.

Families were contacted on a rolling basis until recruitment goals were met throughout the first wave of data collection, which took place over 20 months. Of the 452 eligible children, a random sample of 309 were contacted about participation in the study. Of these 309 families, we were unable to reach and/or schedule 67 families, 15 refused to participate in the study, and 19 were scheduled but failed to show up at their laboratory appointments. The final sample size for the Learning About the Developing Brain Study consisted of 208 children recruited when they were between the ages of 4 and 8 years old, 127 (61%) of which met criteria for an anxiety disorder when they were 2-5 years old.

155 of the 208 children participating in the Learning About the Developing Brain Study were eligible to participate in the first MRI scan in the study. We originally set the age of eligibility for an MRI scan at 6 years old, however, after reasonable success with this age we elected to attempt MRI scans on all children. Following this trial, we found that we could reliably scan children at five and a half years old, which subsequently became our age of eligibility. Of the 53 children who were not eligible to participate in the MRI study, 29 were ineligible because they did not complete the prerequisite mock scanner training before the close of recruitment, 12 exhibited excessive movement during the mock scanner training, 2 were too young to participate during the recruitment period, and 10 had an MRI contraindication (i.e. metal, tubes in ears). Of the 155 eligible children, 56 parents or children refused to participate, 5 did not show up for multiple MRI appointments, and 11 children asked to be removed from the scanner before data could be collected. In total, 83 children completed the first MRI protocol.

Mock Scanning Protocol

Children who came to the laboratory and were eligible for an MRI scan completed a mock scanning training session to assess their willingness to participate in the MRI session and their ability to remain still during the scanning protocol. The mock scanner was located at our child-friendly laboratory where the other assessments (e.g. eye-tracking) were conducted. Our MRI simulation system includes the mock MRI scanner, a goggle-based auditory and visual stimulus presentation and data recording system, and a head-movement monitoring system.

The simulator protocol was designed to teach children the necessity of remaining still during the actual MRI scan and to desensitize them to the sounds and procedures of an MRI scan. When the child first entered the mock scanner suite, they were told that they were going to practice having an MRI. They were informed that the real MRI would take place at the hospital, but told that the real MRI would look and sound very similar to the practice MRI. The child was then informed that MRI machines make loud noises and their primary job was to not move so that their brain picture would not be blurry. They were reminded throughout the mock training session that they needed to stay very still, “like a statue.”

The protocol within the mock scanner took approximately 20 minutes to complete. Following acclimation to the mock scanner and explanation of what to expect, the child was placed in the mock scanner and began a 5-minute “target practice” head motion training game. In this game, the child was told that their goal was to keep their head, which would be depicted as a black cross on a computer screen, within the yellow portion of a target that was also projected on the screen. After the “target practice” game, the child was told that they would be allowed to watch part of a movie, but that they had to continue being still. If the child moved outside the yellow “target zone” at any point during the 10-minute movie clip, the movie was paused and the child was shown the target image again. The movie was not restarted until the child had placed his or her head back into the yellow “target zone.” Finally, the child completed a 5 minute training session on the functional imaging task that they would be completing at the scanner. Sounds associated with MRI acquisition, including fMRI and DTI protocols, were played during both the movie and the fMRI task training phases.

If the child was able to complete the mock scanner training then they were scheduled for a real MRI scan. To re-acclimate the child to the scanning environment the day of their MRI scan, each child completed an additional short mock training session at a separate mock scanning facility located in the hospital adjacent to the actual MRI scanner.

Supplementary References

**1.** Franz L, Angold A, Copeland W, Costello EJ, Towe-Goodman N, Egger H. Preschool anxiety disorders in pediatric primary care: prevalence and comorbidity. *Journal of the American Academy of Child and Adolescent Psychiatry.* Dec 2013;52(12):1294-1303 e1291.

**2.** Egger HL, Erkanli A, Keeler G, Potts E, Walter BK, Angold A. Test-Retest Reliability of the Preschool Age Psychiatric Assessment (PAPA). *Journal of the American Academy of Child and Adolescent Psychiatry.* May 2006;45(5):538-549.

**3.** Achenbach TM, Ruffle TM. The Child Behavior Checklist and related forms for assessing behavioral/emotional problems and competencies. *Pediatrics in review / American Academy of Pediatrics.* Aug 2000;21(8):265-271.

**4.** Goodman R. The Strengths and Difficulties Questionnaire: a research note. *Journal of child psychology and psychiatry, and allied disciplines.* Jul 1997;38(5):581-586.
